# Different therapeutic approaches in melasma: advances and limitations

**DOI:** 10.3389/fphar.2024.1337282

**Published:** 2024-04-02

**Authors:** Parisa Ghasemiyeh, Rahil Fazlinejad, Mohammad Reza Kiafar, Shiva Rasekh, Mohammad Mokhtarzadegan, Soliman Mohammadi-Samani

**Affiliations:** ^1^ Pharmaceutical Sciences Research Center, Shiraz University of Medical Sciences, Shiraz, Iran; ^2^ Department of Pharmaceutics, School of Pharmacy, Shiraz University of Medical Sciences, Shiraz, Iran; ^3^ Department of Material Science and Engineering, Tehran University, Tehran, Iran

**Keywords:** melasma, hyperpigmentation, topical drug delivery, nanoparticles, nanotechnology, skin permeation, clinical effectiveness

## Abstract

Melasma is a chronic hyperpigmentation skin disorder that is more common in the female gender. Although melasma is a multifactorial skin disorder, however, sun-exposure and genetic predisposition are considered as the main etiologic factors in melasma occurrence. Although numerous topical and systemic therapeutic agents and also non-pharmacologic procedural treatments have been considered in melasma management, however, the commonly available therapeutic options have several limitations including the lack of sufficient clinical effectiveness, risk of relapse, and high rate of unwanted adverse drug reactions. Recruitment of nanotechnology for topical drug delivery in melasma management can lead to enhanced skin penetration, targeted drug delivery to the site of action, longer deposition at the targeted area, and limit systemic absorption and therefore systemic availability and adverse drug reactions. In the current review, first of all, the etiology, pathophysiology, and severity classification of melasma have been considered. Then, various pharmacologic and procedural therapeutic options in melasma treatment have been discussed. Afterward, the usage of various types of nanoparticles for the purpose of topical drug delivery for melasma management was considered. In the end, numerous clinical studies and controlled clinical trials on the assessment of the effectiveness of these novel topical formulations in melasma management are summarized.

## 1 Introduction

Melasma is a common hyperpigmentation disorder, especially in the female gender, that is usually presented symmetrically as light brown to dark brown hyperpigmented spots in the face and also it can be exacerbated through prolonged sun exposure (Espósito et al., 2022). Melasma, as a multi-factorial disorder, is the most common pigmentary disorder and can be presented as centrofacial, malar, and mandibular skin patches (Guarneri, 2014) with possible inflammatory features (Noh et al., 2014). The prevalence of melasma can be varied from 1% in the normal population to 50% in the high-risk population. The difference in the prevalence of melasma in different nations can be attributed to ethnicity, genetics, and also the degree of sun exposure. As it has been reported, the prevalence of melasma is much higher in the Middle East and South East Asians, Hispanic Americans, Mediterranean Africans, and also Brazilian (Majid and Aleem, 2021). Melasma can affect all skin phototypes, however, it is more common in the middle (Fitzpatrick skin phototypes II, III, IV, and V) (Guarneri, 2014). The average age of onset of melasma can be varied from 20 to 40 years old according to reports from various nations. In addition, melasma is more predominant in the female gender and it has been reported that females are 9–10 times more prone to melasma disorder in comparison to males (Majid and Aleem, 2021). The prevalence of melasma in females during pregnancy is much higher and can be up to 63% in pregnant women. The most common risk factor for melasma in women is pregnancy, while the most common risk factors in men are sun exposure and positive family history of melasma (Majid and Aleem, 2021). Although melasma is a non-malignant skin disorder, however, if a proper, well-timed, and optimum therapeutic regimen is not considered, it can induce various psychological and emotional distress including feelings of frustration, unattractiveness, and embarrassment (Wu et al., 2021).

## 2 Melasma etiology

Numerous factors have been considered in melasma etiology. In this regard, genetic predisposition and gene polymorphisms, sun exposure and UV radiation, hormonal changes, underlying disorders including thyroid disorders, pregnancy, and also drug-induced melasma have critical roles in melasma induction. A list of drug-induced hyperpigmentation is summarized in Table 1.

**TABLE 1:** A category of drug-induced hyperpigmentation and their pathogenesis.

Pharmacologic category	Drug	Pathogenesis	Ref.
Analgesics	NSAIDs^a^	NSAIDs can act as haptens that can bind to melanocyte-linked proteins and further induction of cytotoxic reaction against the complex and FDE^b^ occurrence which can lead to the post-inflammatory hyperpigmentation	Ben Fadhel et al. (2019), Nahhas et al. (2019)
	Acetaminophen	Acetaminophen can act as hapten that can bind to melanocyte-linked proteins and further induction of cytotoxic reaction against the complex and FDE^b^ occurrence which can lead to the post-inflammatory hyperpigmentation	Fathallah et al. (2011), Genovese et al. (2020)
Cardiovascular drugs	Amiodarone	-Reversible, dose, and time-dependent hyperpigmentation (dose ≥400–800 mg/day and at least 6 months of therapy)	Kounis et al. (1996), Stähli and Schwab (2011)
		-Accumulation of lipofuscin deposits within the dermal histiocytes that can induce hypepigmentation	
		-Acting on lysosomes and induction of phototoxic-induced lysosomal damage	
	Diltiazem	Absorption of UVA and UVB wavelength and induction of photosensitivity reactions	Saladi et al. (2006)
	Eltrombopag	-Stimulation of melanin synthesis	Braunstein et al. (2013)
		-Eltrombopag-induced pigment deposition, inflammation, and vessel damage that can lead to erythrocyte leakage and hemosiderin deposition and therefore hyperpigmentation occurrence	
	Tinzaparin sodium	Unknown mechanism	Takci and Ozoguz (2012), Nahhas et al. (2019)
Antineoplastic agents	5-FU^c^	5-FU intravenous injection can induce the loss of endothelial vessel integrity which can lead to drug leakage from the vessels to the surrounding epidermis which in turn can interfere with the melanogenesis process and might induce hyperpigmentation	Geddes and Cohen (2010), Suvirya et al. (2014)
	Bleomycin	-Irritation, itching, or scratching of the skin followed by drug leakage from the vessels and hyperpigmentation occurrence	Ibrahimi and Anderson (2010), Ziemer et al. (2011)
		-Direct action of bleomycin on keratinocytes and FDE^b^ occurrence	
	Cyclophosphamide	-Genetic predisposition	Chittari et al. (2009)
		-Photosensitivity induction	
		-Focal melanocyte stimulation and therefore pigmentation occurrence	
		-Drug-induced renal dysfunction and further drug accumulation and hyperpigmentation occurrence	
	Ifosfamide	-Genetic predisposition	Reyes-Habito and Roh (2014)
		-Photosensitivity induction	
		-Focal melanocyte stimulation and therefore pigmentation occurrence	
	Daunorubicin	-Photodistribution pattern and skin pigmentation	Kroumpouzos et al. (2002)
		-Stimulation of melanocytes	
	Doxorubicin	-Photodistribution pattern and skin pigmentation	Abbasi and Wang (2008)
		-Stimulation of melanocytes	
	Gefitinib	-Functional changes in melanocytes	Chang et al. (2004)
		-Post-inflammatory process in melanocytes	
		-Enhanced pigment transfer to the keratinocytes and also to the dermal macrophages	
	Hydroxyurea	-Genetic predisposition	Tansir et al. (2022)
		-Focal melanocyte accumulation	
		-Photosensitivity	
	Imatinib mesylate	-Mutation in the *c-kit* gene or variant of the kinase	Di Tullio et al. (2018), Nahhas et al. (2019)
		-Direct effect on melanocytes	
		-Increased pigmentation incidence due to the enhanced drug deposition through the chelation of its metabolite to iron and melanin	
		-Imatinib mesylate-induced immune dysfunction and further melanin incontinence	
	Sorafenib	Drug excretion through the skin	Gupta et al. (2015)
	Sunitinib	-Drug excretion through the skin	Vignand-Courtin et al. (2012)
		-Reversible and dose-dependent hyperpigmentation	
	Paclitaxel	-Local alteration in blood flow and induction of reticulate hyperpigmentation	Cohen (2016)
	Pemetrexed	Unknown mechanism	Schallier et al. (2011)
	Tegaful	-Melanocyte deposition through the direct stimulation of melanocytes via the melanocyte-stimulating hormone	Nahhas et al. (2019)
		-Melanocyte hyperplasia	
		-Decreased keratinocyte turnover	
Antiepileptic agents	Phenytoin	Hydantoin-induced direct stimulation of melanocytes and facial melasma occurrence	Roji et al. (2018)
	Retigabine (Ezogabine)	-Melanin deposition within the perivascular and periadnexal dermal cells	Shkolnik et al. (2014)
		-Tyndall effect	
Antimicrobial agents	Chloroquine/Hydroxychloroquine	-Formation of chloroquine-melanin complex	Mir et al. (2013)
		-Drug-induced hyperpigmented macules merging into facial patches	
	Mefloquine	-Drug-induced hyperpigmented macules merging into facial patches	Nahhas et al. (2019)
	Mepacrine	Drug-induced tissue staining through the acridine dye component of mepacrine	Kleinegger et al. (2000)
	Dapsone	-Photoaggravated hypermelanosis	Garcia and Cohen (2017)
		-Induction of FDE^b^	
	Isoniazid	Enhanced dermal melanophages	KIM et al. (2008)
	Levofloxacin	Direct effect on melanocytes and overproduction of melanin especially in sun-exposed areas and in those with inflammatory skin disorders	Connors et al. (2018)
	Minocycline	Direct effect on melanocytes and overproduction of melanin especially in sun-exposed areas and in those with inflammatory skin disorders	Fiscus et al. (2014)
	Rifampin (Rifampicin)	Drug excretion through the skin	Thangaraju et al. (2015), Nahhas et al. (2019)
	Polymyxin B	Augmentation of melanogenesis pathway through the release of drug-induced inflammatory mediators	Li et al. (2020)
	Emtricitabine	Enhanced melanin of the basal level of the epidermis layer	Nelson and Schiavone (2004)
	Zidovudine	Reversible and dose-dependent hyperpigmentation through the melanocyte stimulation and enhanced melanin amounts	Premjith and Karthikeyan (2022)
Prostaglandin analogs	Latanoprost and Bimatoprost	Enhanced tyrosinase activity and melanogenesis in melanocytes through the prostanoid receptors	Nahhas et al. (2019)
Psychotropic Agents	Chlorpromazine	-UV exposure can lead to the formation of chlorpromazine polymers	Calheiros et al. (2020)
		-Binding of chlorpromazine to melanocytes and induction of melanin synthesis	
	Amitriptyline	Unknown mechanism of pigmentation, however, synergistic hyperpigmentation was seen with concurrent minocycline therapy which induced rapid onset of pigmentation	Nahhas et al. (2019)
	Desipramine	Formation of drug–melanosome complex in response to chronic UV exposure	
	Imipramine	-Formation of drug–melanosome complex in response to chronic UV exposure	
		-Accumulation of imipramine-free radicals	
		-Photoactivation of imipramine and/or its metabolites and induction of tyrosinase activation and increment of melanin synthesis	
Miscellaneous	Adalimumab	Promotion of melanocyte activities and induction of melanin synthesis	Blomberg et al. (2009)
	Afamelanotide	Enhanced eumelanin expression to induce skin pigmentation	De Baat et al. (2020)
	Beta-Carotene, Vitamin A, and Derivatives	Dose dependent skin discoloration	Nahhas et al. (2019)
	Hydroquinone	Skin pigmentation through the local inhibition of homogentisic acid and its metabolites and formation of ochronotic pigments in dermis layer	Mishra et al. (2013)
	Minoxidil	Skin pigmentation through the opening of luminal vessels and reduction of tissue lymphocytes	Nahhas et al. (2019)
	Oral Contraceptives	Estrogen-induced hyperpigmentation and melasma	Resnik (1967)

^a^ Non-steroidal anti-inflammatory drugs.

^b^ Fixed drug eruption.

^c^ 5-Fluorouracil.

As genetics has an important role in the incidence and prevalence of melasma in the patient population, the correlation between various gene polymorphisms and melasma occurrence has been considered. The correlation between Val92Met and Arg163Gln genotypes of Melanocortin-1 Receptor (MC1R) gene and the incidence of melasma was assessed in Indonesian women. In this study, 158 Indonesian women between 18 and 60 years old were included. The Val92Met genotype of the MC1R gene was significantly more common in melasma patients, however, the Arg163Gln genotype was not significantly associated with melasma incidence. In addition, the results of this study showed that the extent of sun exposure and also the positive family history of melasma were another risk factors for melasma occurrence in the Javanese population (Suryaningsih et al., 2019). Another study on the Egyptian population revealed the correlation between vitamin D receptor (VDR) gene polymorphism (TaqI) and melasma incidence. In this regard, 95 Egyptian women were included in this study and results revealed that there was a significant association between t allele and tt genotype with melasma occurrence. Therefore, TaqI polymorphism of the VDR gene was significantly associated with melasma in the Egyptian population (Seleit et al., 2017). Another study shows the correlation between estrogen receptor (ER) gene polymorphisms (PvuII and XbaI polymorphisms for the ERα gene and AluI and RsaI polymorphisms for the ERβ gene) and melasma incidence. In this study, 56 cases and 39 control patients were included. According to the results, there was a positive correlation between the ERα and ERβ genes overexpression and melasma occurrence in the case group. There was a significant association between XbaI polymorphism and melasma incidence. In addition, there was a significant association between AluI and RsaI polymorphisms and melasma. Based on the results patients with Xx, Aa, and RR genotype predominance are more prone to melasma (Bai, 2016).

## 3 Melasma pathophysiology

As mentioned previously, the development of melasma can be affected and precipitated through the various factors including long-term sun-exposure and UV radiation, genetic predisposition, thyroid dysfunction, special drugs, oxidative stress, hormonal imbalance, and female gender especially the pregnant women and also those who are consuming oral contraceptives (OCPs) (Ortonne et al., 2009; Kwon and Park, 2014b; Katiyar and Yadav, 2022). In addition, the activity of various types of skin cells including keratinocytes, mast cells, endothelial cells, fibroblasts, and even sebocytes are associated with melasma incidence (KrupaShankar et al., 2014). Moreover, induction of inflammatory process in the dermis layer and activation of various enzymes including matrix metalloproteinase can cause melasma. Considering the remaining obstacles in melasma treatment, a deeper comprehension of its multifactorial pathogenesis can improve therapeutic outcome and overcome its high recurrence and resistance rates (Rajanala et al., 2019b; Artzi et al., 2021).

During the embryogenic phase, melanoblasts, which are melanocyte precursor cells, migrate, to reach the epidermal layer and hair follicle to create pigments. As listed below, various factors can affect the production of melanin:

a) Production of proopiomelanocortin (POMC) and its derivatives by skin cells.
b) Increased number of melanocortin-1 receptors (MC-1R) on the melanocyte surface.
c) Diacylglycerol (DAG) secretion which activates protein Kinase-C.
d) Nitric oxide (NO) release which triggers cGMP pathways.
e) Increased Cytokine and growth factor production by cytokines.

**FIGURE 1:**
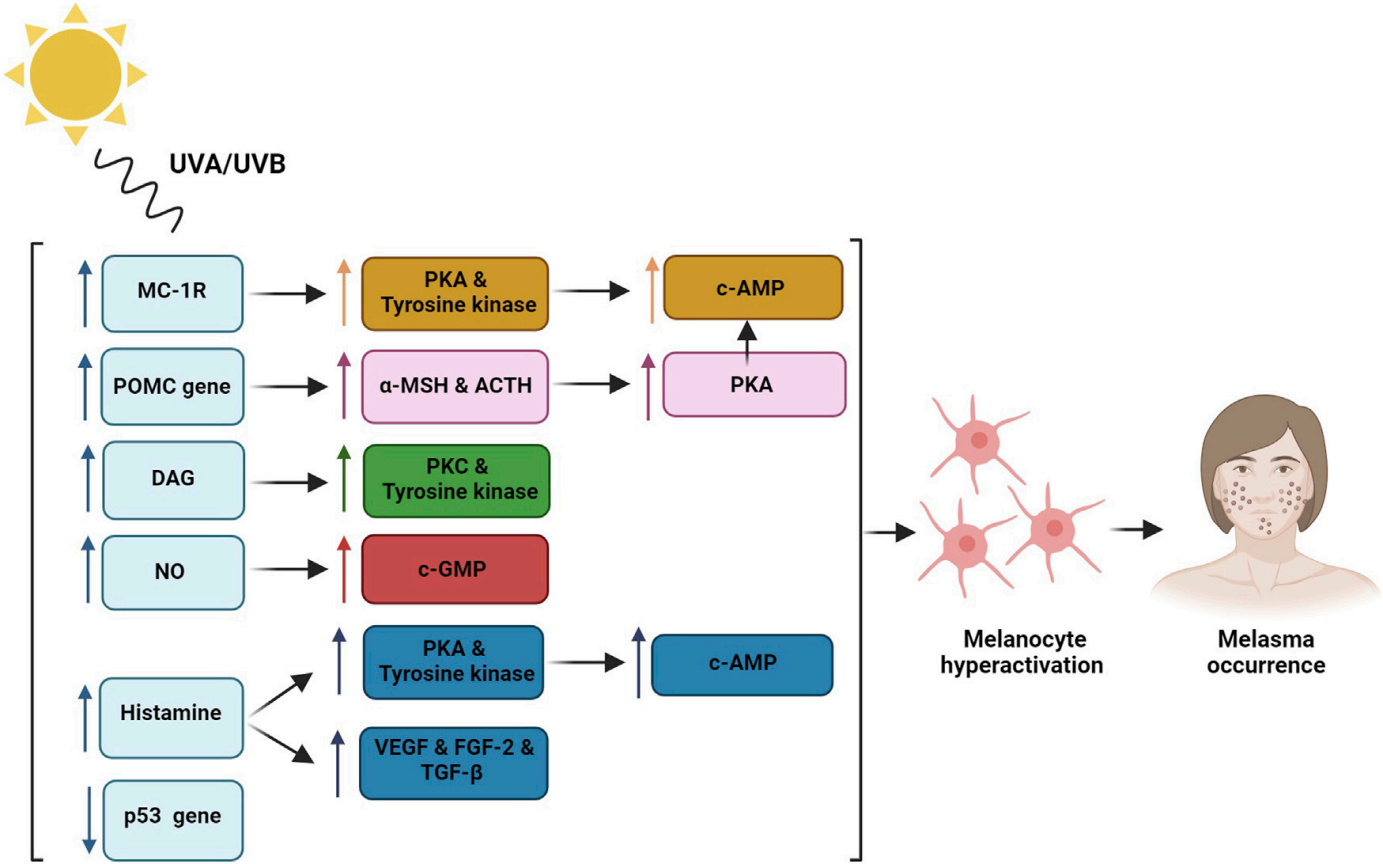
Sun exposure regulates Melanocortin-1 receptor (MC-1R) which induces protein kinase A (PKA) and tyrosine kinase activity that lead to c-AMP phosphorylation and melanocytes hyperactivation; Proopiomelanocortin (POMC) gene induction through the UV radiation enhances alpha-melanocyte stimulating hormone (α-MSH) and adrenocorticotropic hormone (ACTH) production which cause increased PKA and melanogenesis process; Diacylglycerol (DAG) induction through the UV radiation activates protein kinase C (PKC) and tyrosine kinase activity; Nitric oxide (NO) release triggers cGMP pathway; Histamine release from the mast cells activates PKA and tyrosine kinase and induces hyper-vascularization through the induction of vascular endothelial growth factor (VEGF), fibroblast growth factor-2 (FGF-2), and transforming growth factor-beta (TGF-β) that induce melanogenesis process; p53 tumor suppressor gene damage through the UV radiation leads to enhanced melanin production.

Hyperactivity or excess activation of each of these normal pathways can lead to enhanced melanin production in the dermis which in turn can induce irregular hyperpigmentation and melasma occurrence (da Cunha and da Silva Urzedo, 2022). It was shown that prolonged sun-exposure is an important risk factor for melasma development, and can have a crucial role in enhancing each of these melanogenesis paths (Passeron and Picardo, 2018; Artzi et al., 2021). Moreover, recent studies have indicated that the main radiations contributing to melasma incidence are high-energy visible light (HEVL) and long-wave UVA (UVA-1). The combination of these could have a synergic effect causing hyperpigmentation, inflammation, and erythema (Kohli et al., 2018; Kohli et al., 2019). Therefore, melasma can categorize as a photoaging disorder especially in patients with genetic vulnerability (Passeron and Picardo, 2018). The main pathomechanisms of melasma occurrence through the sun-exposure is depicted in Figure 1.

In general, previous studies have found 5 main pathomechanisms associated with melasma occurrence (Kwon et al., 2016; Artzi et al., 2021; Morgado-Carrasco et al., 2022):

### 3.1 Improper melanocytes activity and accumulation of melanocytes in the dermis and epidermis layers

Although increase in melanin levels in melasma patients is a known fact, however, the exact mechanism of this increment is not fully understood. It is known that UV radiation can hyper activate the melanocytes through different ways including the regulation of melatonin-stimulating hormone receptors (MSHR), also known as Melanocortin-1 receptor (MC-1R), which can induce hormone binding and enhanced endogenous protein kinase A (PKA) and tyrosine kinase expression which can in turn lead to the phosphorylation of cAMP and control of melanogenesis that can induce higher melanin production. Moreover, UV radiation can activate the proopiomelanocortin (POMC) gene and thereby enhance the production of alpha-melanocyte stimulating hormone (α-MSH) and adrenocorticotropic hormone (ACTH) which can lead to increased PKA and melanogenesis (Im et al., 2002). Another mechanism would be an elevation in endogenous diacylglycerol (DAG) levels which can activate tyrosine kinase-inducing melanogenesis (Carsberg et al., 1995). In addition, p53 tumor suppressor gene damage through the UV radiation can lead to enhanced melanin production (Videira et al., 2013).

### 3.2 Elevation in mast-cell count and activity

Mast cells are involved in several melasma pathomechanisms. Previous studies have shown that UV radiation can increase histamine release from the mast cells. Histamine binding to H_2_ receptors can activate PKA and induce tyrosinase pathway, and therefore melanogenesis process (Gilchrest et al., 1981; Malaviya et al., 1996; Yoshida et al., 2000). Moreover, histamine can induce melanocyte migration and proliferation (Kim and Lee, 2010). Furthermore, mast cells can cause hyper-vascularization by producing angiogenic factors including vascular endothelial growth factor (VEGF), fibroblast growth factor-2 (FGF-2), and transforming growth factor-beta (TGF-β) (Crivellato et al., 2008). Therefore, mast cells have a crucial role in photoaging caused by prolonged UV exposure and are linked to solar elastosis, vascular dilation, and basal membrane obstruction which are all main features of melasma (Kwon et al., 2016).

### 3.3 Solar elastosis or dermal extracellular matrix abnormality

Chronic exposure to UV radiation can induce unusual accumulation of elastic tissue which can lead to the photoaging process, called solar elastosis. It has been indicated that 83%–93% of melasma patients had varying ranges of solar elastosis including atypical elastic fibers which are thicker, more fragmented, and curled (Kang et al., 2002; Torres-Álvarez et al., 2011). UV radiations can elevate the production of tryptase enzyme in the mast cells. Tryptase in turn can cause solar elastosis by inducing elastin production in fibroblasts (Grimbaldeston et al., 2003). Mast cells can also induce elastin production through the cytokines (Hernández-Barrera et al., 2008) which can lead to the activation of mast cell metalloproteinase (MMP) that in turn induce the degradation of collagen type 4 and damage basement membrane (Bosset et al., 2003; Iddamalgoda et al., 2008).

### 3.4 Hyper-vascularization

In melasma patients 68.75% increase in skin vascularization was obvious in comparison to the normal population (Kim et al., 2007). As mentioned previously, mast cells and keratinocytes can induce vascularization through the increment in VEGF secretion, enhanced TGF-β and FGF-2, thus can create more, larger, and highly dilated vessels which are considered important targets in melasma treatment (Kim et al., 2005; Kwon and Park, 2014a; Kwon et al., 2016). Although VEGF is an important vascularization factor, there is insufficient evidence connecting it to melanogenesis process. Other reasons for hyper-vascularization are UV-induced solar elastosis and elevation of cytokines such as stem cell factor (SCF), inducible nitric oxide synthase (iNOS), and c-KIT (a strong melanogenic cytokine) (Kang et al., 2006; JO et al., 2009). In conclusion, it seems that anti-angiogenic agents would be a promising treatment for melasma (Kwon et al., 2016).

### 3.5 Disruption of the basement membrane

The disruption of the basement membrane is an important finding in melasma. The results of a recent study indicated that 83%–95% basement membrane disruption was obvious in melasma patients through different staining techniques (Torres-Álvarez et al., 2011). Prolonged UV exposure can induce matrix metalloproteinase-2 (MMP-2) and MMP-9 activation in basement membrane which can lead to collagen types 4 and 6 degradation and accumulation of more elastic fibers in melasma patients (Inomata et al., 2003). Moreover, Cadherin-11, an adhesion molecule expressed in fibroblasts, can activate MMP which in turn lead to more collagen deterioration and enhanced elastic fibers in the skin. In addition, Cadherin-11 can activate melanogenesis process in melanocytes (Kim et al., 2016). Furthermore, various factors including aging, iatrogenesis, and the environmental factors can lead to basement membrane damage and therefore easier migration of melanocytes and melanin to skin layers and their accumulation within the dermal layer. These accumulated cells in the dermis layer are called pendulous melanocytes that are usually observed in melasma patients (Kang et al., 2002; Torres-Álvarez et al., 2011). This phenomenon can induce a refractory response in melasma patients that can enhance the possibility of recurrence (Sanchez et al., 1981). In this regard, restoring the basement membrane and preventing the release of melanocytes and melanin into the dermis layer would be essential for long-term treatment of melasma. Consequently, any skin irritation with basement membrane disruption can deteriorate melasma condition and also induce persistence or recurrence type of the disease (Kwon et al., 2016).

### 3.6 Others pathomechanisms

Estrogen can be an important contributing factor in melasma development and its effects are mostly seen in post-puberty and pregnant women, and also in women using oral contraceptives. Moreover, an increase in estrogen and progesterone receptors were obvious in the dermis and epidermis layers of the melasma lesions, respectively. Through the estrogen binding to its receptor, tyrosinase and microphthalmia transcription factor (MITF) can be activated and therefore melanin would be increased (Cohen, 2017). Estrogen can also increase the expression of PDZ domain protein kidney-1 (PDZK-1) which can enhance melanogenesis and melanosomes transfer (Lee, 2015).

Long-term exposure to UV radiation can induce inflammation and enhanced skin fibroblasts which can produce SCF that can bind to its receptor (c-KIT receptor or more specifically m-KIT receptor) and therefore melanin production through the activation of tyrosine kinase paths in melanocytes (Yuan and Jin, 2018; Rajanala et al., 2019b). UV-induced inflammation is commonly caused by the increment in cyclooxygenase-2 (COX-2) and prostaglandin levels which in turn can enhance the tyrosinase pathway and stimulate melanocytes and melanogenesis process. Therefore, it seems that COX inhibitors would be promising drugs to alleviate melasma lesions (Kim et al., 2012; Rajanala et al., 2019b). In addition, an increase in superoxide dismutase and a notable reduction in glutathione levels, as the main cause of oxidative stress, were obvious in melasma patients (Kuthial et al., 2019).

Moreover, through the UVB radiation, the keratinocytes can increase the secretion of cytokines, growth factors, and hormones including iNOS which in turn induces the melanin production in melanocytes (IMOKAWA et al., 1998; Taraz et al., 2017).

It is interesting to know that, despite the whole face sun exposure, only some areas, specifically those with concentrated sebaceous glands including the forehead, cheeks, and upper lips are more commonly affected by melasma. It can be attributed to the ability of these skin regions in vitamin D, different cytokines, and various growth factors production (Abdel-Naser et al., 2012).

## 4 Classification of melasma

Melasma can be clinically classified as centrofacial (with the highest incidence rate), malar and mandibular skin patches. In the centrofacial type, the forehead, cheeks, chin, nose, and upper lips are involved. While in malar melasma, cheeks and nose and in mandibular melasma, mandibular ramus are involved (Guarneri, 2014). In addition, melasma can be classified based on the depth of melanin. In this regard, through Wood’s lamp examination method, melasma has been categorized as epidermal, dermal, mixed, and intermediate types in which the epidermal melasma is the most common type among patients with melasma lesions. However, due to the discrepancies between the Wood’s lamp technique and *in vivo* histopathological results in melasma classification, reflectance confocal microscopy (RCM) was introduced (Liu et al., 2011). Based on the RCM imaging, melasma can be classified to epidermal and mixed types which is more compatible with the histopathological results (Kang and Bahadoran, 2012). RCM technique is a non-invasive, *in vivo* imaging technique that can be used in microscopic skin analysis with high resolution and without the need for skin biopsies preparation (Liu et al., 2011).

## 5 Therapeutic options and limitations

**FIGURE 2:**
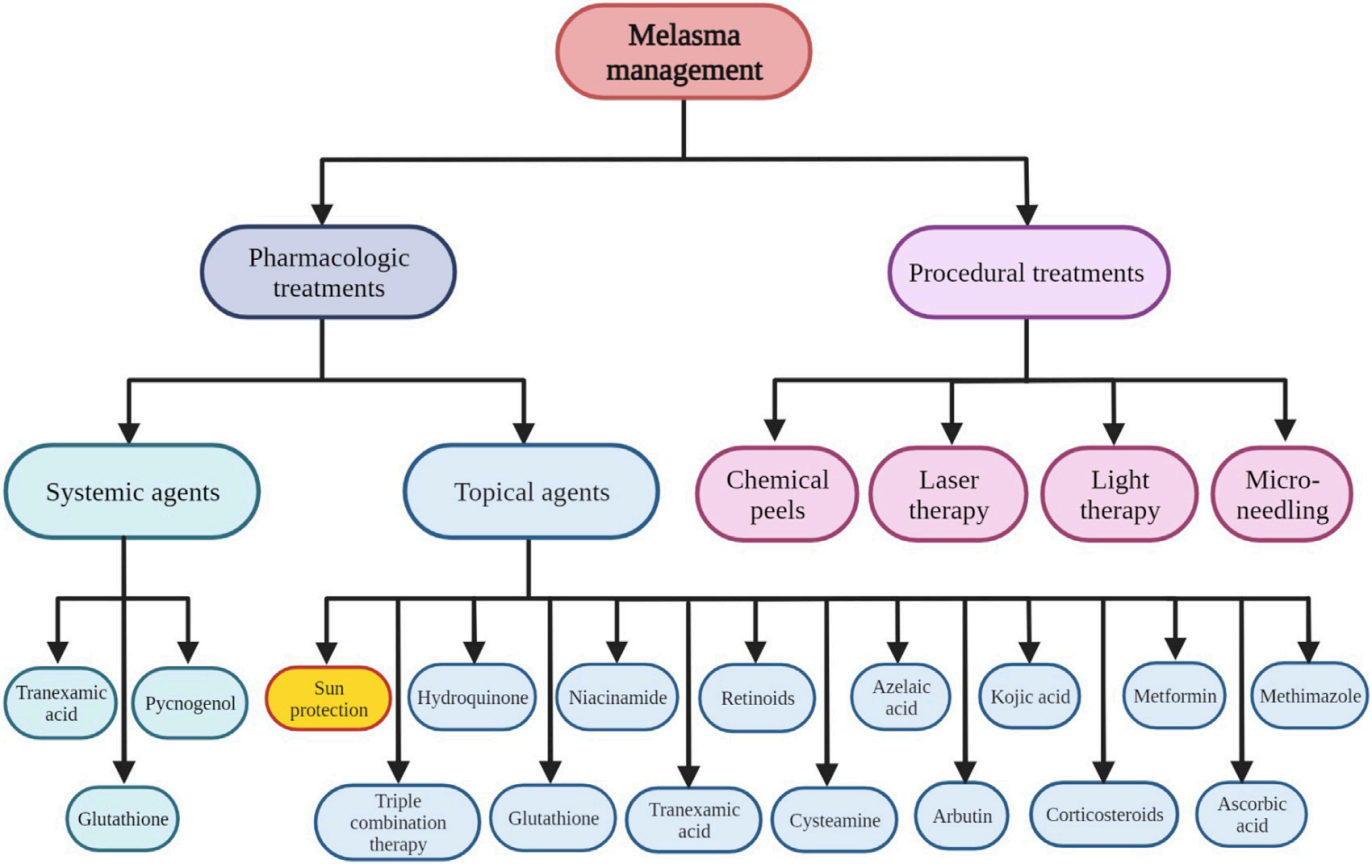
Various pharmacologic and non-pharmacologic therapeutic options considered in melasma management.

As shown in Figure 2, various therapeutic options have been considered in melasma management (Goel and Trivedi, 2023; Li et al., 2023). In this regard, most of them have proceeded through photo-protection, affecting melanocytes’ activity, regulating dermal and epidermal cells including endothelial cells, fibroblasts, mast cells, sebocytes, and keratinocytes which in turn can affect melanocytes activity and reversing abnormal tissue damage and photo aging process (Kwon et al., 2019). Hydroquinone as a topical depigmenting agent is among the most commonly used therapeutic option in melasma management. However, other topical depigmenting agents including niacinamide, azelaic acid, 4-n-butylresorcinol, kojic acid, resveratrol and ascorbic acid are considered in melasma management. In addition, antiaging agents have been added to the depigmenting agents to induce optimum clinical responses. In this regard, topical triple combination therapy containing hydroquinone 4%, tretinoin 0.05%, and fluocinolone 0.01% have received the Food and Drug Administration (FDA) approval in melasma management. Tretinoin have both hypopigmentary and antiaging characteristics. While, steroids including fluocinolone can inhibit inflammation-induced photo damage and melanogenesis. Besides various therapeutic and pharmacological treatment options, numerous procedural treatments including laser therapy, light therapy, and microneedling have been considered in melasma treatment (Kwon et al., 2019).

Although various therapeutic approaches including pharmacological and procedural treatments have been considered in melasma management, however, it is a chronic, recurrent, and relapsing skin disorder. Therefore, melasma treatment, especially in patients with dark skin types classified as Fitzpatrick types IV to VI, is challenging due to the possibility of relapse and also susceptibility to post-inflammatory hyperpigmentation (PIH) (Rodrigues and Pandya, 2015). Various topical and systemic therapeutic agents and also procedural treatments that are considered in melasma management are summarized as follows.

### 5.1 Topical treatments

**FIGURE 3:**
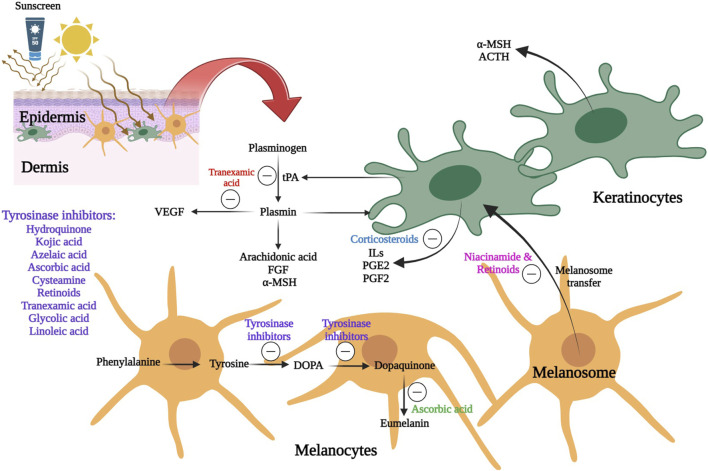
A schematic view of the different therapeutic agents and their mechanism of actions in melasma management 6. Line 562: Review the writing of the SLN abbreviation, since it was previously defined and was already being used as an abbreviation.

Topical route of administration has been considered as the main strategy for melasma treatment. A schematic view of the different therapeutic agents and their mechanism of actions in melasma management is represented in Figure 3.

#### 5.1.1 Sun protections

The key strategy for melasma management is consistent photoprotection through the application of broad-spectrum sunscreens with a notably high level of sun protection factor (SPF) of 50+ and persistent pigment darkening (PPD) of +++ or ++++) (Jansen et al., 2013). Solar elastosis is described as abnormal elastic tissue accumulation within the dermis layer due to the chronic sun exposure or photoaging process. High levels of solar elastosis have been observed in the skin of melasma patients. Furthermore, histological examination revealed that melasma skin is thicker, more curled, and contain more fragmented elastic fibers in comparison to normal skin (Rajanala et al., 2019a). It has been reported that prolonged exposure to both UV and visible light can result in the enhanced pigmentation in all skin types, especially darker ones (Mahmoud et al., 2010). Therefore, one of the most important approaches in melasma management and potentially mitigating its progression is photoprotection. Consequently, regular application of sunscreens along with other therapeutic agents can improved clinical outcome in melasma patients (Lakhdar et al., 2007).

#### 5.1.2 Hydroquinone

Hydroquinone is a hydroxyphenolic natural substance that is widely used in melasma management (Gupta et al., 2006; Tse, 2010). Topical formulations of hydroquinone are commercially available in concentration ranges of 2%–4% and has been recommended as the golden standard for hyperpigmentation disorders including melasma (Boo, 2021). Hydroquinone can disrupt the architecture of melanocytes, and competitively inhibit tyrosinase (Adalatkhah and Sadeghi-Bazargani, 2015; McKesey et al., 2020; Suryantari et al., 2020), thereby it can inhibit the conversion of 1–3,4-dihydroxyphenylalanine to melanin (Tse, 2010) and further induce melanin necrosis, that can avoid the melanogenesis process. Although there are numerous evidences regarding the beneficial effects of topical hydroquinone in melasma treatment (Haddad et al., 2003; Nordlund et al., 2006a; Tirado-Sánchez et al., 2009; Khosravan et al., 2017), however it can induce various adverse reactions including itchiness, erythema, contact dermatitis, and ochronosis (Adalatkhah and Sadeghi-Bazargani, 2015; McKesey et al., 2020; Suryantari et al., 2020). Ochronosis can occur through the prolonged usage of hydroquinone in the absence of sufficient photoprotection (Bronzina et al., 2020). Moreover, leukoderma, permanent depigmentation, vitiligo-like hypochromia (Zhang et al., 2019), and possibility of cancer occurrence can develop due to hydroquinone byproducts (Briganti et al., 2003; Westerhof and Kooyers, 2005). However, the mentioned adverse reactions can be averted through the careful patient monitoring and restricting the duration of hydroquinone therapy (Nordlund et al., 2006b).

#### 5.1.3 Retinoids

All trans retinoic acid (ATRA), isotretinoin, retinol, retinaldehyde, tazarotene, and adapalene are among retinoids used as depigmenting agents in melasma management. They can be administered with the purpose of the inhibition of tyrosinase activity, reduction of melanin transfer, acceleration of keratinocyte turnover, melanin dispersion, and enhanced skin permeation through the stratum corneum layer (Ortonne, 2006). Therefore, retinoids can be used simultaneously with other topical therapeutic agents to enhance their skin permeation (Ortonne, 2006; McKesey et al., 2020).

The application of tretinoin (ATRA) in melasma lesions in concentration ranges of 0.05%–0.1% could effectively decrease skin pigmentation. This depigmenting potential can be attributed to the inhibition of tyrosinase transcription and disruption of melanin synthesis (Rendon and Dryer, 2016; McKesey et al., 2020). Although the efficacy of tretinoin in melasma management has been established, however, a minimum treatment duration of 24 weeks is required to achieve the clinical effectiveness. Furthermore, topical application of retinoids on melasma lesions could potentially be associated with secondary hyperpigmentation as a result of drug-induced irritation (Piętowska et al., 2022). Topical retinoids can also induce erythema, dryness, flaking, and photosensitivity (Ball Arefiev and Hantash, 2012; Chandorkar et al., 2021). Moreover, sensations of burning and stinging were obvious in some patients. Nevertheless, these adverse reactions are predominantly well-tolerated (Rendon and Dryer, 2016; González-Molina et al., 2022).

#### 5.1.4 Corticosteroids

Corticosteroids have a crucial role in inhibition of melanogenesis induced by UV-B radiation. Their potential mechanism of action would be the inhibition of prostaglandins and cytokines, including endothelin 1 and granulocyte macrophage colony-stimulating factor (GM-CSF), which are the main stimulators of melanin production (Ebanks et al., 2009). Corticosteroid as monotherapy depigmenting agents have demonstrated limited effectiveness and also could induce potential adverse reactions including epidermal atrophy, telangiectasia, acne, rosacea-like erythema, and perioral dermatitis (Menter, 2004). Therefore, it would be rational to administer corticosteroids simultaneously with other topical agents for melasma treatment (Menter, 2004; González-Molina et al., 2022).

#### 5.1.5 Triple combination therapy

The clinical effectiveness of triple combination (TC) therapy, also known as Kligman’s-Willi’s formulation, have been demonstrated in melasma patients. A modified triple combination cream consisting of hydroquinone, a retinoid, and a low-potency steroid have been considered as a therapeutic regimen for melasma management. Based on the previous studies, this triple combination therapy is well-tolerated and has sufficient clinical effectiveness. The addition of a steroid to a combination of hydroquinone and ATRA can efficiently suppress the secretory cytokines that are involved in melanocytes activation for melanin synthesis process. Moreover, recruitment of this triple combination therapy regimen can reduce the incidence of inflammation and skin irritation induced by hydroquinone or retinoids (Gupta et al., 2006).

Tri-Luma^®^ cream containing hydroquinone 4%, tretinoin 0.05%, and fluocinolone acetonide 0.1% has been authorized by the FDA for melasma management and should be applied once daily on the affected facial areas for at least 8 weeks (Ferreira Cestari et al., 2007; Spierings, 2020). It has been reported that the variation in clinical effectiveness of triple combination cream and hydroquinone was more obvious in patients with darker skin tones and also in those with mixed type of melasma (Chan et al., 2008).

#### 5.1.6 Azelaic acid

Azelaic acid can inhibit the mitochondrial enzyme and DNA synthesis in melanocytes, which might be cytotoxic. Tyrosinase inhibitory effects of azelaic acid can prevent DNA synthesis in melanoma cell lines without the risk of harmful adverse reactions including ochronosis (Baliña and Graupe, 1991; González-Molina et al., 2022). Previous studies have compared the clinical effectiveness of azelaic acid in comparison to hydroquinone in skin hyperpigmentation management. Based on the reports, azelaic acid was superior to hydroquinone in terms of hyperpigmentation treatment (Baliña and Graupe, 1991; Farshi, 2011a; Komal et al., 2021; Sobhan et al., 2023). Despite the promising therapeutic potential of azelaic acid in melasma, however due to limited water solubility and poor skin permeability, its conventional topical formulations have been fabricated in higher doses (10%–20%) to achieve the desired clinical outcome (Sieber and Hegel, 2014; Sobhan et al., 2023). The most common adverse reactions that have been reported with topical azelaic acid formulations are burning, itching, stinging, dryness, and erythema which are temporary and mild (Kirsch et al., 2019; Malik et al., 2019; Pekmezci, 2019; Searle et al., 2022).

#### 5.1.7 Tranexamic acid

The usage of tranexamic acid in melasma treatment was first documented in 1979. Tranexamic acid is an anti-plasmin substance that reduces the arachidonic acid formation, which in turn lowers the melanocyte-stimulating hormone (MSH) and pigment production (Kanechorn et al., 2012b). Moreover, tranexamic acid can inhibit the pigmentation induced by sun exposure and UV radiation (Shihab et al., 2020). Furthermore, it has been shown that endothelin-1 and VEGF, which are responsible for enhanced vascularity in melasma lesions, can be diminished by tranexamic acid administration. Tranexamic acid can be administered through either oral, intradermal, or topical route for melasma treatment (Shankar et al., 2014; Adalatkhah and Sadeghi-Bazargani, 2015; McKesey et al., 2020; Suryantari et al., 2020).

Administration of topical azelaic acid 20% and tranexamic acid 5% showed promising results in post-inflammatory hyperpigmentation management in patients diagnosed with acne vulgaris. Nevertheless, it seems that topical tranexamic acid would be safer in comparison to azelaic acid for hyperpigmentation treatment (Sobhan et al., 2023).

#### 5.1.8 Kojic acid

Kojic acid can be effective in melasma management through the inhibition of free tyrosinase synthesis (VIEIRA BRAZIL et al., 2023). Kojic dipalmitate which is the esterified derivative of kojic acid can underwent *in situ* hydrolysis in the different skin layers to release kojic acid. The released kojic acid can further inhibit the tyrosinase and melanin synthesis process. The main advantages of kojic dipalmitate over kojic acid would be its photostability, thermal stability, and stability at a wide range of pH in various topical formulations. However, due to the crystallinity nature, high lipophilicity, and low water solubility of kojic dipalmitate, its incorporations within the topical formulations would be more challenging (Zilles et al., 2023). Based on the previous studies, a combination of topical kojic acid and hydroquinone would be a promising depigmenting agent regimen. Kojic acid can also be used in contact dermatitis and erythema (Deo et al., 2013; Yenny, 2018). Previous studies have reported the favorable clinical efficacy outcomes of different concentrations of topical kojic acid formulations either alone or in conjunction with other therapeutic agents (Monteiro et al., 2013; Saeedi et al., 2019).

#### 5.1.9 Cysteamine

Cysteamine is a biosynthetic aminothiol that produced in mammalian cells, is widely known for its antioxidant properties (Besouw et al., 2013). In addition, numerous *in vivo* and *in vitro* studies have demonstrated its anti-carcinogenic and anti-mutagenic properties. Furthermore, cysteamine has been recognized as an efficient depigmenting agent for melasma treatment (Besouw et al., 2013). Although the exact mechanism of depigmenting potential of cysteamine is unclear, however, it has been reported that melanocytotoxicity is not involved (Qiu et al., 2000; Karrabi et al., 2021). In spite of promising results regarding the topical cysteamine therapy for melasma in terms of clinical efficacy and safety, however, its application is challenging due to its instability and also its unpleasant odor produced during the oxidation process (2013).

#### 5.1.10 Ascorbic acid

Ascorbic acid, also known as vitamin C, is an antioxidant that can bind to copper and successfully inhibit the tyrosinase enzyme. Therefore, it can suppress the oxidative polymerization of melanin intermediates. Consequently, melanin production in the melanogenesis process would be inhibited through the ascorbic acid administration (Ebanks et al., 2009; Telang, 2013). Based on the previous studies, ascorbic acid, as a depigmenting agent, could be well-tolerated with minimal risk of irritation in comparison to topical hydroquinone 4% (Espinal-Perez et al., 2004; Hwang et al., 2009). Various topical formulations of vitamin C with concentration range of 3.75%–30% have been considered in melasma, however, in most studies, vitamin C concentration was less than 10% for the purpose of skin photodamage and melasma treatment (Correia and Magina, 2023).

#### 5.1.11 Glycolic acid

Glycolic acid, as an α-hydroxy acid (AHA), have a crucial rule in cell-adhesion disruption and further skin desquamation. In addition, glycolic acid can inhibit tyrosinase activity and therefore suppress the melanin production (Chaudhary and Dayal, 2013; Austin et al., 2019). Numerous studies have indicated that glycolic acid peels can augment the clinical effectiveness of other topical agents, particularly in individuals with darker skin tones (Ibrahim et al., 2015; Choi et al., 2019). The most common adverse reactions of topical glycolic acid would be mild to moderate erythema, pruritus, and inflammation. Administration of intense moisturizing agents would be helpful to alleviate these unfavorable adverse reactions (Ibrahim et al., 2015).

#### 5.1.12 Niacinamide

Niacinamide, also known as vitamin B3, is the active form of niacin (Farshi, 2011b; Rolfe, 2014) and can be used in melasma (Navarrete-Solís et al., 2011) and hyperpigmentation (Kimball et al., 2010) disorders through the regulation of melanosomes transfer from the melanocytes to the keratinocytes. Therefore, niacinamide can diminish melanin accumulation in the skin layers. In addition to its depigmenting potential, niacinamide showed anti-inflammatory and photoprotective characteristics against the solar degenerative alterations (Farshi, 2011b; Rolfe, 2014). In this regard, administration of topical niacinamide has been widely considered in melasma management. Prolonged use of topical niacinamide might be accompanied by some mild adverse reactions including mild burning, erythema, and pruritus (Taylor et al., 2003).

#### 5.1.13 Salicylic acid

Salicylic acid, as a β-hydroxy acid (BHA), is commonly utilized in cosmetic products as a peeling agent for skin lightening purposes. This effect can be attributed to its keratolytic potential and its ability to dissolve lipids. In addition, salicylic acid has antibacterial and anti-inflammatory characteristics. Administration of topical salicylic acid in melasma treatment can result in some adverse reactions including erythema, burning sensation, irritation, peeling, blistering, or crusting (Dahl et al., 2013; González-Molina et al., 2022). The efficacy of salicylic acid peels in melasma treatment can be enhanced through the combination therapy with mandelic acid (Spierings, 2020).

#### 5.1.14 Arbutin

Arbutin is an organic glucopyranoside that can inhibit tyrosinase activity and avoid melanocyte maturation without the risk of toxic effects. In addition, deoxy-arbutin is a dose-dependent tyrosine hydroxylase inhibitor which can further inhibit the melanogenesis process. Therefore, topical arbutin with skin-lightening potential would be promising for melasma and hyperpigmentation treatment (Raton, 2011; Searle et al., 2020).

#### 5.1.15 Other topical agents

Other topical agents including thiamidol, linoleic acid, phytic acid, yeast extract, mulberry extract, rucinol, undecylenoyl phenylalanine, and epidermal growth factors have been reported in previous studies as effective compounds in melasma management (Lee et al., 2002; Gupta et al., 2006; Khemis et al., 2007; Alvin et al., 2011; Katoulis et al., 2014; Lyons et al., 2018; Huerth et al., 2019; González-Molina et al., 2022).

### 5.2 Oral treatment

#### 5.2.1 Tranexamic acid

Oral tranexamic acid has been shown to be effective as an adjuvant therapy for refractory cases of melasma or as a second or third-line of treatment (Kim et al., 2017). Tranexamic acid is usually administered in melasma at a dosage of 250 mg twice daily (Shin et al., 2013) either monotherapy or in combination with other therapeutic options (Karn et al., 2012; Kim et al., 2017; Bala et al., 2018). Numerous clinical trials have indicated that depigmenting effect of tranexamic acid was observed following a therapeutic course of at least 2–3 months (Lee et al., 2016a; Kim et al., 2017). The duration of tranexamic acid therapy should not be shortened due to the high risk of melasma relapse after discontinuation (Kim et al., 2017). The possible adverse reactions reported with oral tranexamic acid therapy are abdominal bloating, headache, and menstrual irregularities that are rare and transient (Lee et al., 2016a). The main concern regarding systemic tranexamic acid therapy would be the risk of thromboembolic events. In this regard, precise screenings for personal and family history of thromboembolic events, stroke, and heart disease should be considered prior to therapy initiation (Ball Arefiev and Hantash, 2012; Lee et al., 2016a).

### 5.3 Miscellaneous agents

#### 5.3.1 Glutathione

Glutathione is a biosynthetic tripeptide consisting of glutamate, cysteine, and glycine, which is considered as one of the most potent endogenous antioxidants. The mechanism of skin lightening of glutathione can be attributed to the inhibition of tyrosinase and further alteration in the transformation of eumelanin to pheomelanin (Pillaiyar et al., 2017; Weschawalit et al., 2017; Grimes et al., 2019).

#### 5.3.2 Carotenoids

Carotenoids are naturally occurring pigments extracted from plants, algae, and photosynthetic bacteria. They are known with their anti-inflammatory, antioxidant, and photoprotective properties that can avert the photo aging process (Galasso et al., 2017).

#### 5.3.3 Thiamidol

Thiamidol is a potent tyrosinase inhibitor that can efficiently prevent UVB-induced hyperpigmentation (Vachiramon et al., 2021). The results of a randomized clinical trial revealed that the efficacy of thiamidol cream 0.2% was comparable with hydroquinone cream 4% (Vachiramon et al., 2021).

#### 5.3.4 Antioxidants

Various antioxidants including ascorbic acid and zinc have been frequently administered through the topical or oral routes for melasma management (Sarkar et al., 2012b; Yousefi et al., 2014). The application of topical vitamin C and zinc resulted in a notable amelioration of skin lesions among with minimal adverse reactions (Sharquie et al., 2008; Hwang et al., 2009). Other antioxidants including Korean red ginseng (Song et al., 2011), *Petroselinum Crispum* (Khosravan et al., 2017), and orchid extracts were also considered in melasma treatment and showed favorable efficacy and tolerability (Tadokoro et al., 2010).

#### 5.3.5 Pycnogenol

Pycnogenol is a standardized herbal extract with high bioavailability, synergistic effects with other lightening agents, and low toxicity potential through the oral route of administration. Pycnogenol was resulted in reduced hyperpigmentation in melasma patients after 1 month of systemic therapy (Sarkar et al., 2012b; Babbush et al., 2021).

### 5.4 Procedural treatments

#### 5.4.1 Chemical peels

Chemical peels including glycolic acid, salicylic acid, or trichloroacetic acid have been considered in melasma management and have shown moderate clinical efficacy. However, chemical peels may potentially induce irritation, burning, and inflammation following treatment, and also may cause melasma relapse (Sheth and Pandya, 2011; Sarkar et al., 2012a).Glycolic acid is the most commonly used chemical peel for melasma treatment (Erbil et al., 2007). Based on the previous studies, either monotherapy or combination therapy of glycolic acid with hydroquinone and tretinoin was not associated with superior clinical outcomes and could induce more adverse reactions (Lim and Tham, 1997; Faghihi et al., 2011; Chaudhary and Dayal, 2013).

#### 5.4.2 Laser and light-based therapies

Laser and light-based therapies are modalities that are utilizing light energy to treat skin lesions (Piętowska et al., 2022). Laser and light therapy are considered as a third-line of therapeutic options in melasma management for those who are unresponsive to topical therapeutic agents and chemical peels. Laser and light therapies can accelerate the elimination of melanin (Trivedi et al., 2017). Intense pulsed light (IPL), Low-fluence Q-switched (LFQS) lasers, non-ablative fractional lasers (NAFL), and picosecond lasers are among the most frequently used light and laser-based therapies in melasma management (Goel et al., 2011). However, their response might be unpredictable and result in relapse hyperpigmentation (Hofbauer Parra et al., 2016; Piętowska et al., 2022).

#### 5.4.3 Micro-needling

Micro-needling procedure, which is commonly considered as a collagen induction treatment, involves repeated skin puncturing with sterile microneedles (Ball Arefiev and Hantash, 2012). This procedure can elicit a physiological reaction that can further facilitate the wound repairment process and collagen and elastin synthesis (Bailey et al., 2022). Micro-needling can be used to augment the dermal and transdermal delivery of active pharmaceuticals. Micro-needling can preserve the integrity of the epidermis layer while it can accelerate the healing process and reduce the risk of infection and scar formation (Cohen and Elbuluk, 2016; Saleh et al., 2019).

## 6 New approaches in melasma pharmacotherapy

Various adverse drug reactions are associated with systemic treatment and also numerous challenges are existing regarding the skin penetration and clinical efficacy of the topical conventional formulations that are considered in melasma management. In this regard, in the recent years, new approaches including the recruitment of nanotechnology in targeted topical drug delivery have been considered to overcome these drawbacks and cause optimum clinical response (Salvioni et al., 2021) Therefore, different types of nanoparticles including lipid nanoparticles, nanoemulsion/microemulsion, vesicular nanocarriers, polymeric nanoparticles, nanocrystals, and metal nanoparticles have been used as topical drug delivery systems for melasma management.

### 6.1 Lipid nanoparticles

Solid lipid nanoparticles (SLNs) and nanostructured lipid carriers (NLCs), as the first and second generations of lipid nanoparticles, respectively, have promising characteristics for topical drug delivery purposes. In addition, SLNs and NLCs can accompany superior cosmetic and dermatological benefits, including increased skin elasticity, enhanced hydration, improved skin penetration and drug deposition, and drug protection against degradation (Hajare et al., 2014; Ghanbarzadeh et al., 2015b).

#### 6.1.1 SLNs

SLNs are colloidal drug delivery system that are consisted of lipid matrices that are solid at body temperature and also emulsifiers. These nanoparticles typically have an average diameter between 50 and 1,000 nm (Paliwal et al., 2020).

SLNs showed promising results for encapsulation of hydroquinone as a hydrophilic agent. In this regard, hydroquinone encapsulation within the SLNs was accompanied by higher drug stability against the oxidation process and enhanced skin penetration along with diminished systemic absorption. Results of previous studies on topical gel formulation of hydroquinone-loaded SLNs indicated a significantly higher drug deposition within the epidermis layer (46.5% ± 2.6%) in comparison to the conventional gel of hydroquinone (15.1% ± 1.8%) (Ghanbarzadeh et al., 2015b). In addition, the results of an *in vitro* permeation study on rat skin revealed that drug accumulation within the skin layers was approximately 3 times higher, while the drug flux into the receptor phase of Franz cells was about 6.5 times lower in hydroquinone-loaded SLNs in comparison to the hydroquinone gels which confirmed the reduced systemic absorption and further reduced adverse drug reactions through the encapsulation in SLNs (Wu et al., 2017).

Kojic acid encapsulation within the SLNs could significantly improve its dermal delivery. In this regard, kojic acid-loaded SLNs showed higher drug concentration within the skin layers, controlled drug release, and greater tyrosinase inhibition capability was seen in comparison to the conventional formulation (Khezri et al., 2020b).

#### 6.1.2 NLCs

Based on the results of previous studies, hydroquinone encapsulation within the NLCs was accompanied by enhanced drug stability, targeted drug delivery, and diminished skin irritation (Wu et al., 2019). Moreover, hydroquinone-loaded NLCs significantly improved skin penetration and UVA/UVB radiation protection in comparison to the hydroquinone conventional formulation (Wu et al., 2017).

According to the previous studies, azelaic acid-loaded NLCs was resulted in more occlusive properties, enhanced skin permeation, targeted drug delivery to the melanocytes with enhanced clinical efficacy (Kumari et al., 2015). Moreover, the sustained release capability of azelaic acid-loaded NLCs would be advantageous for topical drug delivery due to the prolonged localized drug deposition within the skin layers (Tangau et al., 2022).

### 6.2 Liposomes/nanosomes

Liposomes are vesicular nanoparticulate delivery systems that are composed of lipid bilayers of phospholipids and cholesterol. Liposomes are capable of encapsulating both hydrophobic and hydrophilic drugs and also have the potential of fusion with the cell membrane to modulate its fluidity and facilitate the skin penetration and distribution of the loaded drug (Sharma et al., 2018). Recruitment of liposomes as topical drug delivery systems would be promising due to their numerous advantages including enhanced skin penetration through the stratum corneum layer, skin moisturizing and restoring effects, controlled drug release, and biocompatibility and biodegradability properties (Rahimpour and Hamishehkar, 2012).

Results of a previous *in vitro* study indicated that arbutin-loaded liposomes had a slower drug flux and absorption rate along with higher and longer skin deposition in comparison to arbutin solution. Consequently, systemic absorption of the loaded drug was significantly reduced through the encapsulation within the liposomes (Wen et al., 2006b).

Patients with melasma were treated using liposomal serum that contained azelaic acid, 4-n-butylresorcinol, and retinol. Following the treatment course, the MASI score of the included patients was increased from 41.7% to 85%, however, the melasma severity scale (MSS) was decreased from moderate (score 2) to mild (score 1) during the treatment course (Kusumawardani et al., 2019).

The findings of a preliminary investigation indicated that liposomal hydroquinone, as a tyrosinase inhibitor, effectively enhance the therapeutic efficacy. Hydroquinone 4%-loaded niosomes were fabricated through the fusion method and characterized. The obtained MASI scores from this plot study indicated appreciable therapeutic effectiveness of liposomal hydroquinone in comparison to the conventional hydroquinone cream (Banihashemi et al., 2015a). Results of another study indicated that although the therapeutic effect of hydroquinone in melasma treatment was preserved after encapsulation in liposomes, however, no significant superiority was observed in comparison to the conventional cream (Taghavi et al., 2019b).

The incorporation of 4-n-butyl resorcinol into liposomes was resulted in enhanced drug stability, improved skin permeation, as well as increased tyrosinase inhibitory potential which in turns resulted in more efficient melanogenesis inhibition (Huh et al., 2010).

### 6.3 Niosomes

Niosomes are vesicular nanocarriers that are composed of nonionic surfactants with permeation enhancing potential. Niosomes are fabricated through the self-aggregation of nonionic surfactants in an aqueous environment usually through the either thin film hydration or solvent injection techniques (Rigano and Lionetti, 2016; Singh and Sharma, 2016).

Kojic acid and hydroquinone were simultaneously encapsulated within the noisomes. The prepared topical formulation exhibited prolonged drug release patterns (Divanbeygikermani et al., 2018).

### 6.4 Transfersomes

Transfersomes are biocompatible vesicular nanocarriers with high deformability potential that are composed of lipids bilayer and membrane softeners (Chiranjeevi et al., 2013; Fadel et al., 2017). Transfersomes are promising in transdermal drug delivery due to their high deformability potential, ease of skin permeation through the stratum corneum layer, enhanced transepidermal drug flux, and longer skin deposition (Hatem et al., 2018).

The incorporation of niacinamide into the transfersomes was accompanied by enhanced skin permeation and improved depigmenting efficacy in comparison to the conventional liposomal niacinamide formulation (Lee et al., 2016b).

Arbutin encapsulation within the transfersomes was resulted in augmented skin permeation and enhanced depigmenting effectiveness of the loaded drug (Wen et al., 2006b).

### 6.5 Nanoemulsions/microemulsions

Nanoemulsions are considered as suitable drug delivery systems to pass through the lipophilic barriers for dermal delivery purposes. Nanoemulsion and microemulsions are composed immiscible aqueous and organic phases that are stabilized through the incorporation of relatively larger amounts of suitable surfactants as emulsifiers. Nano and microemulsions are promising in the field of cosmeceutics and topical drug delivery systems with the main advantage of enhanced drug solubility, improve skin penetration through the stratum corneum barrier, and enhanced bioavailability (Ghosh and Murthy, 2006; Hatem et al., 2020).

Arbutin w/o/w nanoemulsions that were co-encapsulated with coumaric acid showed enhanced encapsulation efficiency, increased drug stability, sustained drug release, and improved skin delivery in comparison to the free drug (Wen et al., 2006a; Huang et al., 2019). Moreover, arbutin was co-encapsulated with lactic acid and niacinamide within the microemulsions in order to enhance drug stability, skin permeation, and whitening effect (Surini and Mellani, 2017b).

Results of a previous study on azelaic acid and hyaluronic acid-loaded nanoemulsion showed superior drug deposition within the skin layers, enhanced tyrosinase inhibition potential, and reduced cytotoxicity (Tomić et al., 2019b). Moreover, addition of hyaluronic acid to the azelaic acid nanoemulsions efficiently reduce melanin synthesis through the enhanced melanocyte-nanoemulsion interactions (Atrux-Tallau et al., 2014b).

Based on the results of a previous *in vitro* study, hydroquinone 4% microemulsions showed higher amounts of drug release, higher drug stability, and reduced skin irritation and skin layer disturbance in comparison to the conventional hydroquinone cream (Üstündağ Okur et al., 2019). Moreover, hydroquinone encapsulation within the microemulsion was accompanied by augmentation in skin permeability through the stratum corneum and also enhanced photostability potential (Tirnaksiz et al., 2012; Salimi and Hajiani, 2018).

Kojic monooleate, as a known tyrosinase inhibitor, was encapsulated in nanoemulsion and cytotoxicity results revealed a survival rate of 54.76% for 3T3 cells (Syed Azhar et al., 2018). Furthermore, kojic dipalmitate encapsulation within the nanoemulsions was accompanied by improved drug stability (Al-Edresi and Baie, 2009).

The incorporation of ascorbic acid within the microemulsions efficiently improve skin permeation of the loaded drug and also induce higher skin protection capabilities (Pakpayat et al., 2009).

Co-encapsulation of kojic acid and arbutin within the microemulasions was accompanied by higher photostability potential against the UVB radiation for both drugs in comparison to the aqueous solution of kojic acid and arbutin (Gallarate et al., 2004).

### 6.6 Metal nanoparticles

Gold nanoparticles dispersion in water with great stability, biocompatibility, and chemical inertness potential are suitable nanocarriers for topical drug delivery purposes (Khodakiya et al., 2012).

Arbutin and gold nanoparticles were mixed to fabricate a nanocomplex with higher skin lightening potential. The results of this study indicated that the prepared arbutin nanocomplex was accompanied by lower intracellular and extracellular melanin production, higher anti-inflammatory effects, and reduced toxicity potential in comparison to the free drug (Jiménez-Pérez et al., 2018; Park et al., 2019a).

### 6.7 Polymeric nanoparticles

Polymeric nanoparticles including nanospheres and nanocapsules can act as a matrix or reservoir system (Banihashemi et al., 2015b). Therefore, they can be used to encapsulate various therapeutic agents to control the drug release pattern and extend the drug deposition within the skin layers for topical delivery purposes (Guterres et al., 2007).

Arbutin-loaded cross-linked amphiphilic guar gum nanoparticles were prepared and characterized. Based on the results, the prepared polymeric nanoparticles had a higher degree of hydrophobicity which lead to the improved skin penetration through the stratum corneum. In addition, the results of cytotoxicity on human keratinocyte (HaCaT cells) showed lower toxicity potential for the prepared arbutin-loaded polymeric nanoparticles (Bostanudin et al., 2021).

The application of N-(2-hydroxyl) propyl-3-trimethyl ammonium chitosan chloride as a liposome surface coating material was accompanied by enhanced kojic acid skin permeation and reduced melanin synthesis in comparison to the conventional liposomal kojic acid formulation (Wang et al., 2012a; Singh et al., 2023).

Encapsulation of ascorbic acid in ethyl cellulose nanoparticles was resulted in enhanced drug stability, increased anti-tyrosinase activity, and therefore improved skin whitening potential (Duarah et al., 2017b; Singh et al., 2023).

### 6.8 Nanocrystals

Nanocrystals with improved drug solubility, enhanced dissolution rate, and increased skin adhesiveness are promising for topical drug delivery purposes (Vidlářová et al., 2016; Malamatari et al., 2018).

Azelaic acid is a water soluble drug with two carboxylic acid groups in its structure, therefore, it has limited skin permeability. In this regard, fabrication of azelaic acid nanocrystals dispersed in Pluronic F127 and hyaluronic acid was accompanied by enhanced solubility, dissolution rate, drug stability, and skin permeability (Tomić et al., 2019b).

### 6.9 Fullerenes

Fullerenes, also known as C60 carbon nanotube cylinder, are spherical nanoparticles with carbon in their structure (Jiménez-Pérez et al., 2018). The huge interior volume of the fullerenes are capable to load various biomolecules. In addition, the external surface of the fullerenes can be chemically modified for targeted topical drug delivery purposes (Koo et al., 2005).

In another study L-ascorbic acid and arbutin-loaded fullerenes incorporated within polyvinyl pyrrolidone. According to the results this delivery system were associated with enhanced anti-tyrosinase activity and reduced UVA-induced melanogenesis in comparison to L-ascorbic acid and arbutin solution (Xiao et al., 2007b).

In general, although topical treatment would be promising in melasma management, however, most of the available conventional topical formulations are challenging due to the limited skin permeability, low water solubility, low dermal bioavailability, and therefore insufficient clinical response. Therefore, as shown in Table 2, various types of nanoparticles including nanoemulsions/microemulsions, lipid nanoparticles, vesicular nanocarriers, polymeric nanoparticles, and gold nanoparticles were utilized to enhance skin permeation, improve drug solubility, increase photostability, control the drug release profile, induce longer drug deposition within the skin layers, and therefore enhance the clinical effectiveness of the therapeutic options for melasma treatment.

**TABLE 2:** A summary of different types of nanoparticles, the loaded cargo, particle size, and advantages of drug encapsulation in melsma management.

Nanocarrier	Drug	Drug percentage	Particle size	Entrapment efficiency (%EE) and loading capacity (%LC)	Advantages/Outcome	Reference(s)
SLNs^a^	Hydroquinone	2%	86 nm	%EE: 89.50% ± 4.50%	• Enhanced skin deposition	Ghanbarzadeh et al. (2015a), Salimi and Hajiani (2018)
					• Higher drug accumulation within skin layers	
				%LC: 11.20% ± 1.30%		
					• Reduced systemic absorption	
	Kojic acid	0.2%	156.97 ± 7.15 nm	%EE: 59.02% ± 0.74%	• Improve dermal delivery of kojic acid	Khezri et al., 2020a
				%LC: 14.75% ± 1.63%		
NLCs^b^	Hydroquinone	5%	393.30 ± 28.23 nm	%EE: 22.13% ± 2.66%	• Enhanced drug stability	Wu et al. (2017)
					• Diminished skin irritation	
				LC: 19.28% ± 4.77%	• Improved skin penetration	
					• Enhanced protection against UVA/UVB radiation	
	Azelaic acid	NA^c^	81.57 ± 9.6 nm	NA	• Targeted drug delivery to the melanocytes	Kumari et al. (2015)
					• Improved effectiveness	
					• Delayed drug release	
					• Reduced adverse drug reactions effects due to the gradual exposure of the skin with lower concentrations of azelaic acid	
Liposomes	Azelaic acid & 4-n-butylresorcinol and retinol	NA	NA	NA	• Reduced melasma severity	Kusumawardani et al. (2019)
	Hydroquinone	4%	126 nm	NA	• Preserved therapeutic effectiveness	Taghavi et al. (2019a)
	Azelaic acid	20%	500 nm	%EE: 85.73%	• Maintain therapeutic efficacy	Ayumi et al., 2019; Akl (2022b), Pasca et al. (2022)
					• Lower recurrence rate	
					• Fewer adverse reactions	
	Kojic acid and hydroquinone	NA	10 µm	NA	• High encapsulation efficiency	Divanbeygikermani et al. (2018), Kusumawardani et al. (2019)
					• High protection against photodegradation and oxidation of hydroquinone	
					• Prolonged drug release pattern	
	Arbutin and coumaric acid	0.05% arbutin and 0.05% coumaric acid	569.67 nm	%EE: 91.08% for arbutin and 80.92% for coumaric acid	• Improved drug stability	Taghavi et al. (2019a), Huang et al. (2019)
					• Enhanced drug solubility	
					• Sustained drug release	
	Arbutin	4%	179.9–212.8 nm	%EE: 17.6% ± 1.38%	• Maintain therapeutic efficacy	Wen et al. (2006b)
	Tranexamic acid	5%	126 nm	NA	• A significant reduction in melasma area and severity index	Banihashemi et al. (2015b)
					• No serious adverse drug reaction	
					Resulted in omparative clinical responses to hydroquinone 4% as an standard medication for melasma management	
Niosomes	Kojic acid and hydroquinone	NA	<10 µm	NA	• Prolonged drug release pattern	Divanbeygikermani et al. (2018)
	Arbutin	0.5%	114.76 nm	%EE: 35.55% ± 1.59%	• High encapsulation efficiency of arbutin within niosomes	Radmard et al. (2021)
					• Enhanced *in vivo* skin permeation and topical delivery along with reduced transdermal delivery in comparison to arbutin plain gel	
					• No potential toxicity and high cell viability percentage of about 86%	
					• No skin irritation potentail	
Nanoemulsions	Arbutin and coumaric acid	NA	Pore diameter of 1–50 µm	NA	• Zero-order drug release pattern	Huang et al. (2019)
	Azelaic acid	1%	419 nm	%EE: 84.65%	• Enhanced skin penetration	Jacobus Berlitz et al. (2019)
					• Decreased tyrosinase activity	
					• Improve skin permeation and targeted delivery to dermis and epidermis	
					• No cytotoxicity potential	
					• Promising for dermal melasma management	
	Licorce	1%	62.7 nm	NA	• Enhanced whitening effect	Atrux-Tallau et al. (2014a)
					• Enhanced epidermal and dermal bioavailability	
					Enhanced *in vitro* cellular uptake	
Microemulaion	Ascorbic acid	4%	<100 nm	NA	• Improved skin permeation	Pakpayat et al. (2009)
					• Enhanced skin protection against UV radiation	
					• Targeted delivery to the epidermis and dermis layers	
					• Promising for melasma management and relieve of oxygen matrix damage	
	Alpha arbutin, lactic acid, and niacinamide	NA	<100 nm	NA	• Enhanced drug stability	Surini and Mellani (2017a)
					• Concurrent administration of three active ingredients with various mechanism of actions for melasma management	
	Hydroquinone	4%	358 nm	NA	• Reduced skin irritation or epidermal layer disturbance	Üstündağ Okur et al. (2019)
					• Enhanced skin permeation through the stratum corneum	
					• Enhanced *in vitro* drug release	
					• Enhanced photostability of the loaded drug	
	Kojic acid and arbutin	0.25% kojic acid & 0.25% arbutin	25–30 nm	NA	• Enhanced photostability of the loaded drugs	Gallarate et al. (2004)
					• The presence of linalool in the prepared formulation could enhance kojic acid photostability	
Gold nanoparticles	Arbutin	0.5%	10.30–17.13 nm	NA	• Enhanced anti-inflammatory properties	Park et al., 2019b
					• Improved bioavailability	
					• Significantly reduced intracellular and extracellular melanin content	
					• Increases tyrosinase enzyme inhibition	
					• Reduced arbutin-related toxicities	
Polymeric nanoparticles	Kojic acid	10 µM	441 nm	%EE: 3.6%		Wang et al. (2012b)
	Azelaic acid	10%	38.3–117.7 nm	NA	• Significant reduction in melanin synthesis	Tomić et al. (2019a)
					• Improved skin diffusivity	
					• Improved skin bioavailability	
					• Enhanced water solubility and dissolution rate	
	Ascorbic acid	50 mg	209–260 nm	%EE: 69%–96%	• Sustained drug release within 8 h	Duarah et al. (2017a)
					• Enhanced *ex vivo* skin permeation	
Nanocrystals	Azelaic acid	10%	38.3–117.7 nm	NA	• Improved skin diffusivity	Tomić et al. (2019a)
					• Improved skin bioavailability	
					• Enhanced water solubility and dissolution rate	
Transfersomes	Ascorbic palmitate	∼13%	110 nm	%EE: 91.3%	• Enhanced drug penetration and deposition within the epidermis layer	Wen et al. (2006b), Li et al. (2021)
				%LC: 11.9%	• Sustained drug release	
					• Reduced skin irritation	
	Linoleic acid	0.05% and 0.1%	151.2 nm and 237.2 nm	%EE: 23.55 ± 5.29 and 62.64 ± 5.49	• Enhanced stability of the loaded linoleic acid	Celia et al. (2012)
					• Increased penetration through the stratum corneum layer	
Fullerenes	L-ascorbic acid and arbutin	50 µM	NA	NA	• Diminished UVA-induced melanogenesis	Xiao et al. (2007a), Xiao et al. (2007b)
					• Reduced melanin synthesis	

^a^ Solid lipid nanoparticles.

^b^ Nanostructured lipid carriers.

^c^ Data not available.

## 7 Clinical outcomes of pharmacotherapy

To date, numerous clinical studies including double- or single-blinded randomized clinical trials and split-face studies have been conducted to assess the therapeutic effect of various topical conventional and novel formulations in melasma management as summarized in Table 3.

**TABLE 3:** A summary of clinical trials outcomes of different topical formulations in melasma management.

Type of clinical study	Therapeutic option	Number of cases	Clinical outcome	Reference
Single-blinded comparative study	Topical Vitamin C Nanosomes	14	• Better clinical response in comparison to glycolic acid 70% chemical peel	Sobhi and Sobhi (2012)
			• Considered as safe with no severe adverse drug reaction	
Uncontrolled study	Topical tranexamic acid emulsion	25	• Successful results in 80% of participants during 8-week period	Kondou et al. (2007)
			• No observed adverse drug reactions	
Split-face trial	Topical liposomal tranexamic acid	23	• A noteworthy reduction in mMASI score after 12 weeks of treatment in comparison to the baseline	Banihashemi et al. (2015b)
Prospective, randomized, single-blind study	Topical Politranexamide liposomal emulsion	26	• A significant improvement in melasma lesions and reduction in MASI score after 6 and 12 weeks of treatment	Manfreda et al. (2023)
Double-blind randomized clinical trial study	Topical hydroquinone-loaded NLCs^a^	20	• A statistically significant reduction in mMASI score	Taghavi et al. (2019a)
Visual assessment	Topical niacinamide-loaded flexible liposomes	21	• A statistically significant increase in the whitening efficacy after 4 and 8 weeks of therapy compared to the baseline	Lee et al. (2016b)
Comparative, randomized, controlled study	Topical liposomal azelaic acid cream	50	• Superior clinical efficacy of liposomal azelaic acid 20% in comparison to hydroquinone 4%	Akl (2022a)
			• More tolerable in comparison to hydroquinone cream	
			• Improved MELASQOL	
Prospective comparative split-face study	Topical arbutin-loaded chitosan nanoparticles	20	• Better therapeutic efficacy for arbutin-loaded chitosan nanoparticles in comparison to the conventional arbutin hydrogel	Hatem et al. (2022)
			• Reduced mMASI score and reduced epidermal melanin particle size surface area (MPSA) in comparison to the conventional arbutin hydrogel	
Randomized, double-blind, vehicle-controlled, split-face study	4-n-butyl resorcinol and resveratrol-loaded liposomes	21	• A significant reduction in melanin index (MI) of the melasma lesions	Kwon et al. (2020)

^a^ Nanostructured lipid carriers.

### 7.1 Tranexamic acid

Topical liposomal tranexamic acid was designed and developed to minimize skin irritation and enhance whitening effects. Numerous studies have demonstrated that liposomal tranexamic acid was effective in approximately 80% of participants during an 8-week of treatment course (Manosroi et al., 2002). However, the results of a trial conducted in Thailand in 2012, showed that liposomal tranexamic acid had no additional benefit compared to conventional tranexamic acid formulation that would be due to the small sample size of this study (Kanechorn et al., 2012a). Nonetheless, the results of this study confirmed the clinical effectiveness of topical tranexamic acid with a significant reduction in average MASI scores after 12 weeks of treatment. Interestingly, these positive effects were persistent even 1 month after therapy discontinuation (Kanechorn et al., 2012a).

A previous study compared the efficacy and tolerability of two liposomal emulsions including Politranexamide^®^ (sample A) and acetylglucosamine, ethyl linoleate, and phenylethylresorcinol (sample B) in facial melasma treatment. Based on the results of this report sample A was superior compared to sample B in melasma treatment after 6 and 12 weeks of patient monitoring. Moreover, Both treatment groups showed a noteworthy reduction in MASI score from the baseline (Manfreda et al., 2023).

### 7.2 Hydroquinone

A clinical study was conducted to assess the therapeutic efficacy of liposomal hydroquinone 4% in melasma management. The patients were randomly allocated to the conventional hydroquinone 4% and liposomal hydroquinone 4% groups and the melasma severity was assessed through the MASI score at defined time points. The findings of the study indicated that although the liposomal hydroquinone 4% was efficient in melasma management and could induce a significant reduction in MASI score, however, no superiority was obvious in comparison to the conventional hydroquinone cream (Taghavi et al., 2019a).

### 7.3 Azelaic acid, 4-n- butyl resorcinol, and retinol

The clinical effectiveness of a liposomal serum containing azelaic acid, 4-n-butylresorcinol, and retinol was assessed in patients with malar pattern melasma. Following treatment, the MSS was improved from moderate to mild, and both the MASI score and the melasma quality of life scale (MELASQOL) had a significant improvement at the end of the therapy in these patients. Therefore, it seems that the prepared topical liposomal preparation was promising in melasma treatment (Kusumawardani et al., 2019).

### 7.4 Aloe vera

The results of a double-blind, randomized clinical trial on melasma patients revealed a 32% improvement in the MASI score for the liposomal aloe vera gel group, while only a 10% improvement was obvious for the conventional aloe vera gel group (Ghafarzadeh and Eatemadi, 2017).

### 7.5 Vitamin C

The safety and efficacy of topical vitamin C nanosome with iontophoresis was evaluated through a single-blind clinical study and results were compared with 70% glycolic acid chemical peel in patients with melasma. The results revealed that the vitamin C nanosome was superior to the glycolic acid peel hyperpigmentation management. Furthermore, vitamin C nanosomes was accompanied by reduced incidence of adverse drug reactions including skin burning, irritation, and dryness (Sobhi and Sobhi, 2012).

### 7.6 4-n-butylresorcinol and resveratrol

The results of a previous clinical study on liposomal 4-n-butyl resorcinol and resveratrol cream showed superior efficacy in melasma management. This liposomal formulation was accompanied by a significant reduction in melanin index (MI) of melasma lesions after 2 weeks of treatment, while the alteration in MI in the normal skin was not significant (Kwon et al., 2020; Shaw et al., 2022).

### 7.7 Arbutin

A prospective comparative split-face study was conducted in patients with melasma to assess the clinical efficacy of arbutin-loaded chitosan nanoparticles hydrogel in comparison to conventional arbutin gels during a 2-month treatment course. The results of this study revealed a better therapeutic efficacy for arbutin-loaded chitosan nanoparticles in terms of reduction in MASI score and reduction in epidermal melanin particle size surface area (MPSA) in comparison to the free drug (Hatem et al., 2022).

### 7.8 Niacinamide

A clinical study was designed to assess the efficacy of niacinamide-loaded flexible liposomes with skin whitening potential in patients with melasma. M-values, as a measure of melanin estimated using the Mexameter, were assessed after 4 and 8 weeks of therapy initiation. The results revealed a significant improvement in M-values, 9.96% and 16.80% after 4 and 8 weeks of therapy, respectively, compared to the baseline values. Moreover, the visual assessment results and subjective evaluations reports revealed a statistically significant increase in the whitening efficacy of niacinamide after 4 and 8 weeks of therapy compared to the baseline (Lee et al., 2016b).

### 7.9 Azelaic acid

Another clinical study was conducted in females with melasma to assess the effectiveness of topical liposomal azelaic acid 20% cream in comparison to the conventional hydroquinone 4% cream during a 3-month treatment course. All participants were also received once daily oral doses of tranexamic acid during the treatment course. According to this study results a pronounced improvement in patients who were received liposomal azelaic acid cream in comparison to the conventional hydroquinone cream were seen. Moreover, the tolerability of liposomal azelaic acid was significantly superior to hydroquinone cream (Hagag and Allah, 2022).

## 8 Conclusion

In conclusion, melasma is a chronic skin disorder with numerous etiologies including sun exposure and geographic environment, genetic predisposition, and also drug-induced and disease-induced hyperpigmentary skin disorders. Although various therapeutic modality including systemic and topical pharmacological and procedural treatments are commonly available for melasma management, however, many of these treatments showed limited clinical response and accompany numerous unwanted adverse drug reactions. Moreover, due to the nature of melasma and other hyperpigmentary skin disorders, relapse and recurrence after therapy discontinuation is very common. Therefore, design and development of efficient topical therapeutic agents would be promising in melasma management not only to induce optimum clinical response, but also avoid unwanted adverse drug reactions and relapse through the enhance in water solubility, increase the skin permeability, augment the photostability, and improve the dermal bioavailability of the loaded drugs.
